# Tumor‐infiltrating lymphocyte transfusion in a patient with treatment refractory triple negative breast cancer

**DOI:** 10.1002/cnr2.1894

**Published:** 2023-09-26

**Authors:** Arielle Jacover, Yonaton Zarbiv, Keidar Haran Tal, Shira Klein, Shani Breuer, Ronen Durst, Batia Avni, Sigal Grisariu, Polina Stepensky, Michal Lotem, Ofra Maimon, Tamar Yablonski‐Peretz

**Affiliations:** ^1^ Hadassah Medical Center Sharett Institute of Oncology Jerusalem Israel; ^2^ Faculty of Medicine The Hebrew University of Jerusalem Jerusalem Israel; ^3^ Hadassah Medical Center Hadassah Cancer Research Institute Jerusalem Israel; ^4^ Department of Pathology Hadassah Medical Center Jerusalem Israel; ^5^ Department of Cardiology Hadassah Medical Center Jerusalem Israel; ^6^ Department of Bone‐Marrow Transplant Hadassah Medical Center Jerusalem Israel

**Keywords:** adoptive cell therapy, triple negative breast cancer, tumor infiltrating lymphocyte

## Abstract

**Background:**

Triple negative breast cancer (TNBC) is an aggressive form of breast cancer that is treated with chemotherapy. Recently, programmed death 1 (PD1) inhibition, as well as antibody‐drug conjugates, have been added to the available treatment regimen, yet metastatic disease is fatal. Adoptive cell therapy (ACT) using tumor infiltrating lymphocytes (TILs) has been well described in melanoma, but less data is available on other solid malignancies.

**Case:**

Herein, we present a case of a 31‐year‐old patient diagnosed with Breast Cancer gene 1 (BRCA1) positive, TNBC. The patient's disease rapidly progressed while under standard treatment protocols. As a result, additional genetic testing of the tumor was carried out and revealed loss of BRCA1 heterozygosity, a double Tumor Protein 53 (TP53) mutation, and MYC amplification. Due to resistance to conventional therapy, an experimental approach was attempted using tumor‐infiltrating lymphocytes in November 2021 at Hadassah University Medical Center. While receiving this treatment, the patient exhibited a reported subjective clinical improvement including a month spent out of the hospital. However, the final result, presumably due to Interleukin 2 (IL‐2) toxicity, was the patient's passing.

**Conclusion:**

This case is unique and peculiar regarding the treatment modality chosen, due to the extremely refractory disease the patient suffered from. After standard therapies rapidly failed, adoptive cell therapy was attempted with the infusion of TILs. This treatment has been shown effective in melanoma, however, there is an extreme paucity of data on other solid tumors, including TNBC. Although the patient ultimately demised presumably due to treatment side effects, brief clinical benefit was apparent. Further studies are warranted.

## 1 INTRODUCTION

Prior preclinical and clinical studies have already shown that tumors from patients with advanced melanoma contain tumor‐infiltrating lymphocytes with antitumor reactivity targeting a variety of melanoma‐associated antigens. Less data is available for non‐melanoma solid tumors.

These tumor infiltrating lymphocytes (TILs) can be expanded ex vivo using interleukin‐2 (IL‐2) with or without OKT‐3 antibody stimulation and can cause regression of melanoma when adoptively transferred to the patient. A pre‐condition to the success of the transfer is the administration of lymph‐depleting chemotherapy prior to the adoptive transfer of the TILs which greatly enhances their antitumor effect. For example, in a single institution Phase II study, using a preparative non‐myeloablative regimen of Cyclophosphamide and Fludarabine was associated with a 51% objective response rate to TILs and IL‐2. Some complete and durable responses were noted in this 35‐patient population that had been heavily pretreated.^1^ Significantly, a recent phase III study was recently published in the New England Journal showing the efficiency of TIL therapy relative to cytotoxic T‐lymphocyte‐associated protein 4 (CTLA4) inhibition in refractory melanoma.^2^

In melanoma, laboratory studies have shown that the molecular targets of these TILs were frequently shared differentiation proteins associated with melanin synthesis and production. For example, melanoma antigen recognized by T cells 1 (MART‐1), tyrosinase, gp100, or epitopes unique to the autologous tumor, presumably mutated proteins are alleged to be targeted by TILs. The presumption that TILs may target neo‐antigens not specific to melanoma has created interest in adoptive cell therapies in other solid tumors.^3^ Rosenberg et al showed that TILs can target Kirsten rat sarcoma virus (KRAS), Mucin 4, Cell Surface Associated (MUC4), and SMAD Family Member 5 (SMAD5) in epithelial cancers using an in vitro model.^3^ Significantly, Rosenberg's team successfully administered TILs reactive against mutant versions of four proteins—Solute Carrier Family 3 Member 2 (SLC3A2), KIAA0368, Calcium Dependent Secretion Activator 2 (CADPS2), and Cathepsin B (CTSB), in patients with refractory triple‐negative breast cancer (TNBC).^4^ However, there are very few reports of attempted TIL therapy in this type of cancer.

The entry to the clinic of monoclonal antibodies blocking the programmed death‐1 (PD‐1) receptor changed the landscape for cancer immunotherapy. Clinically significant tumor regression was achieved with minimal toxicity, illustrating the potential of checkpoint modulators to leverage immunotherapy. The possible synergistic effect of checkpoint inhibition with adoptive cell therapy (ACT) is of particular interest.^3^

With the advent of immune checkpoint inhibitors, it quickly became evident that a subset of tumor types respond to PD‐1 inhibition producing a durable response. This suggested that, following checkpoint blockade, the adaptive immune system was responsible for the antitumor response.^5^

It was shown that an abundance of the neo‐antigens present in tumors may predict response to checkpoint blockade in both murine models^6^ and human tissue.^7^

Tumors harboring mutations in DNA mismatch repair proteins have been shown to possess a prototypical defect associated with high tumor mutational burden and therefore a wealth of mutation‐associated neoantigens.^8^

Here, we describe a case of a patient with chemotherapy‐resistant triple‐TNBC, harboring a pathogenic Breast Cancer gene 1 (BRCA1) mutation in the tumor. Although the patient was treated with anti‐PD‐1 therapy as well as poly ADP ribose polymerase enzyme (PARP) inhibition, tumor progression was rapid. This patient received adoptive cell therapy using her autologous TILs with the production of a significant, yet short‐term response to this treatment. Significantly, this study reports a treatment modality with very little data regarding this type of malignancy.

## 2 CASE

**FIGURE 1:**
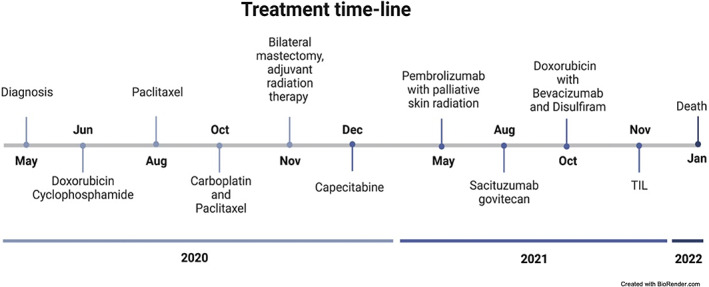
Treatments and interventions the patient received over time.

A 31‐year‐old woman presented with locally advanced breast cancer, diagnosed after self‐discovery of a lump in her right breast. The patient was treated at the Sharret Institute of Oncology, Hadassah University Medical Center in Jerusalem, Israel between May 2020 and June 2022 (see Figure 1 for the treatment timeline). Upon diagnosis, imaging (mammogram, ultrasound, breast MRI, and PET‐CT) demonstrated two distinct tumors in the right breast, with marked right axillary lymphadenopathy and no evidence of distant disease.

Biopsy from the tumor was positive for mixed Invasive ductal carcinoma–invasive lobular carcinoma (IDC‐ILC) histology, estrogen receptor‐negative, progesterone receptor‐positive (50%), and human epidermal growth factor receptor 2 (HER‐2) negative. Germinal evaluation revealed pathogenic frameshift polymorphism in the BRCA1 gene (c.68_69delAG; p.Glu23Val*f*s*17). Neoadjuvant treatment with Doxorubicin and Cyclophosphamide resulted in a major clinical and radiographical response reducing tumor size from 8 to 2 cm per MRI and marked diminution of axillary lymphadenopathy. As per international guidelines, four rounds of Doxorubicin and Cyclophosphamide (protocol AC) were administered due to dose‐limiting toxicity. Subsequently, there was only a minor clinical response with Paclitaxel, therefore concomitant Carboplatin was added. The addition of Carboplatin did not seem to augment the treatment response.

**FIGURE 2:**
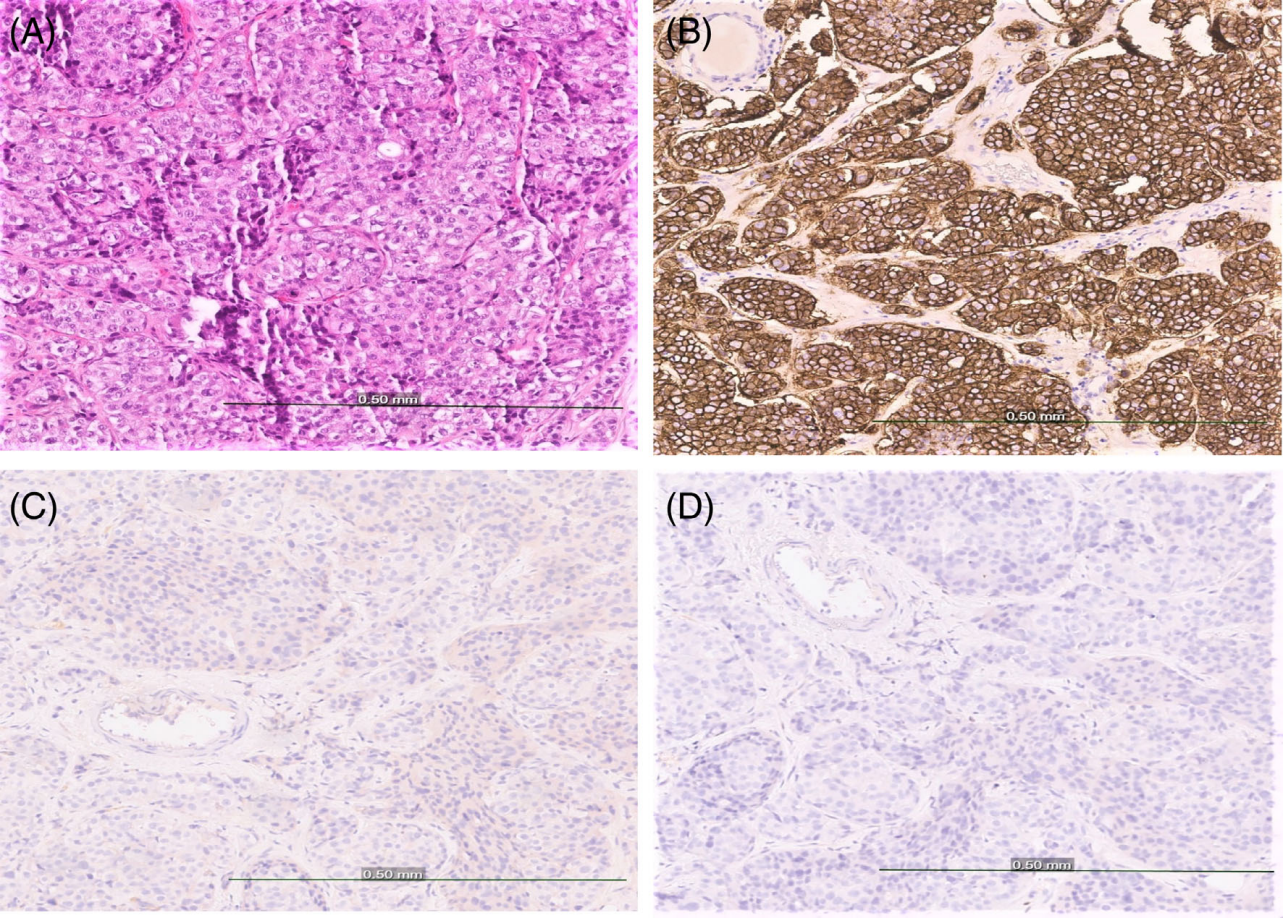
Immunohistochemical staining of tumor cells. (A) H&E control shows invasive ductal carcinoma grade 3 (B) HER‐2 immunostaining control. (C) HER‐2 immunostaining of tumor cells was negative. (D) ER receptor immunostaining was negative (Scale bar = 0.5 mm, ×200 magnification).

**FIGURE 3:**
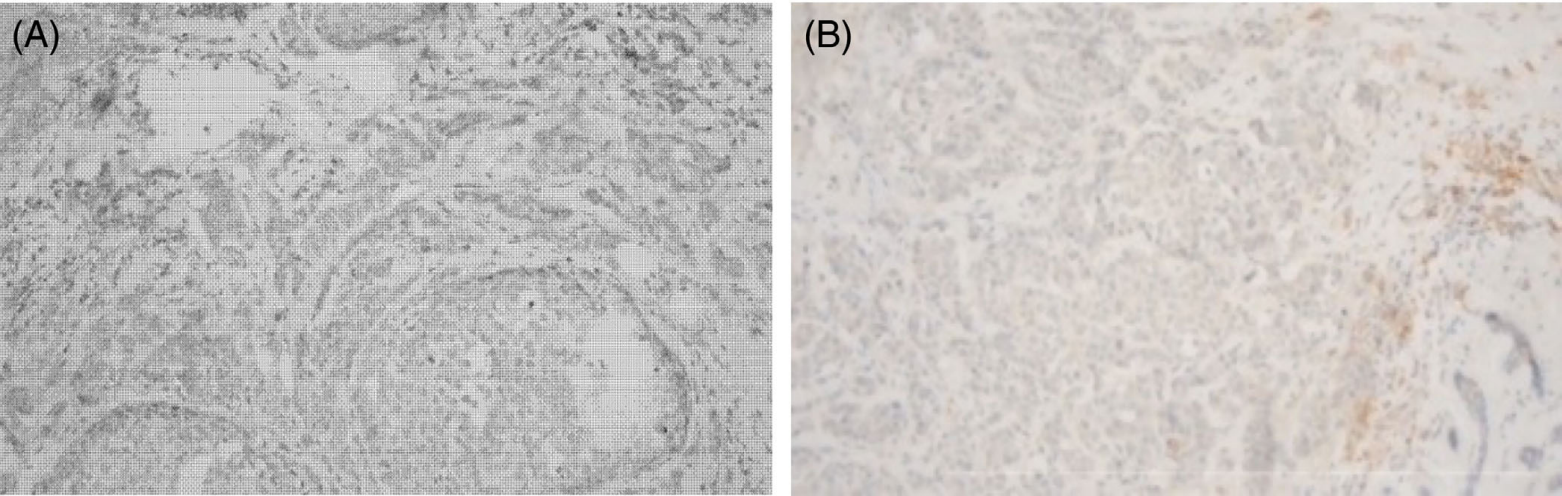
PD‐L1 expression in tumor‐associated immune cells. (A) Staining of PD‐L1 using Optiview DAB detection Kit (Roche) shows no staining in tumor cells. (B) PD‐L1 immunohistochemical stain (Clone 22C3) on the tumor. No PD‐L1 expression in tumor cells. PD‐L1 expression in tumor‐associated immune cells (ICS: 5%; bar marks 500 μ, ×200).

The patient underwent a bilateral mastectomy, pathology revealed a 3.5 cm residual mass with 7/8 positive lymph nodes. Immunohistochemistry showed TNBC (Figure 2) with a KI67 index of 60%. Staining of tumor cells for programmed death‐ligand 1 (PD‐L1) was borderline (Figure 3).

The patient underwent adjuvant radiation therapy to the chest wall and axilla. Soon after the completion of radiation, additional skin and lung nodules were observed and Capecitabine was initiated. There was some disease stabilization, followed by progressive disease. Consequently, Olaparib treatment was begun due to the pathogenic polymorphism in the BRCA1 gene. Upon further progression, a skin biopsy was taken and genomic tumor analysis was performed with the following results: loss of the wildtype BRCA1 allele in the tumor, amplification of MYC (complete gene, non‐focal, >5 copies), and a double Tumor Protein 53 (TP53) missense mutation (c.818G>A; p.Arg273His).

Pembrolizumab was started concomitantly with palliative skin radiation. Initially, disease stabilization was reported, followed by further progression. We, therefore, attempted treatment with Sacituzumab govitecan. Two months later, the skin condition deteriorated and disease progression involving the lungs and skeleton was evident in follow‐up radiographical studies. Doxorubicin rechallenge together with Bevacizumab and Disulfiram was prescribed.

Due to the continuation of disease progression, a treatment course of tumor‐infiltrating lymphocytes was proposed. After obtaining informed consent, a previously described protocol used at our institution was initiated. Briefly, tumor tissue was taken by biopsy from a skin mass of the patient, and TILs were extracted and expanded in vitro with IL‐2.

After a week of expansion, 10 × 10^6^ cells were generated. In parallel, the patient received a protocol of lymphodepletion with Cyclophosphamide, 3000 mg/day for 2 days, and Fludarabine, 45 mg/day for 5 days. Of note, Olaparib was re‐initiated. To ensure TILs persistence and incorporation into the tumor, IL‐2 treatment was administered in a continuous infusion to a total of 18 million IU per 24 h. However, IL‐2 was halted due to hypotensive shock and renal failure after only a total of 45 million units. Four days after the TILs administration the patient was transferred to the intensive care unit with the diagnosis of a life‐threatening cytokine release storm, as a result, a hydrocortisone regimen was appointed. The patient exhibited new onset heart failure with severely reduced left ventricular systolic function, renal failure, and pulmonary congestion. Given that both troponin and proBNP were elevated she was diagnosed with acute myocarditis. Cardiac function improved with conventional heart failure and steroid treatment.

During this time, remarkably a reduction in tumor burden was noticed mostly in the skin and the patient reported subjective relief. The patient's condition improved, and she was discharged from the hospital for 1 month.

Clinically, the patient improved subsequent to hospital discharge and on frequent follow‐up appointments. However, most probably as a side effect of the steroid treatment, the disease began a quick and fatal relapse, presumably from dampening the TIL antitumor effect. Unfortunately, this caused the patient's demise 1 month after the TILs and IL‐2 therapy commenced.

To the best of our knowledge, this case represents one of the few reported treatments utilizing ACT with TIL infusion in solid tumors other than melanoma and specifically TNBC.

## 3 DISCUSSION

We describe herein a patient with TNBC, with a genomic BRCA1 pathogenic polymorphism, together with loss of heterozygote in the tumor, MYC amplification, and a double missense TP53 mutation. Interestingly, the arginine residue replaced in this TP53 mutation is markedly conserved among species. In silico analysis predicts this type of mutation results in the loss of function of the gene. This amino acid is evolutionary conserved and located at the DNA binding domain of TP53. According to bio‐molecular models, it appears as very resistant to treatment and has been reported mostly in individuals suffering from Li‐Fraumeni and Li‐Fraumeni‐like syndromes.^9^, ^10^, ^11^ This TP53 mutation is most known to be a somatic one but has also been described as a germline mutation.^10^ Furthermore, MYC amplification together with the gene expression signature of MEK activity has been associated with resistance to chemotherapy, especially in TNBC.^12^

Presumably, due to the tumor molecular profile, the tumor was extremely resistant to standard chemotherapy protocols, as well as biological, immunological, and ADC (antibody‐drug conjugate) drugs. Therefore, an experimental approach for this type of cancer was suggested. Autologous TILs together with the administration of IL‐2 are established practices in other types of cancer such as melanoma. Here, we showed that for BRCA1 positive, TNBC, this protocol resulted in a reported subjective improvement and objective clinical stabilization including 28 days out of the hospital. Unfortunately, the clinical benefit was short‐term, supposedly due to IL‐2 toxicity and the necessity of immunosuppression with high‐dose corticosteroids. Another possibility of the short‐term benefit may be the inherent difference in immune biology between UV‐induced neoantigen presentation and response to TIL therapy, relative to BRCA‐driven TNBC which is presumably less amenable to this treatment. Regretfully, the end result was the patient's death. We suggest evaluating potential resistance to conventional anti‐cancer therapies based on somatic tumor evaluation and considering initiating experimental potentially active therapy at an earlier stage of disease progression.

Given the paucity of data on TIL therapy for solid tumors, especially in chemotherapy refractory TNBC, we consider this case to be exceptional and uncommon. As far as we know, there are no existing clinical studies investigating this approach specifically in TNBCs, making this case particularly noteworthy. We believe that the unique treatment approach, in this case, could be of interest to practitioners who encounter similar refractory TNBC cases. However, due to the lack of sufficient data, further studies in animal models and phase 1 trials are needed to explore the potential of adoptive cell therapies, including autologous TILs, in breast cancer. Additionally, determining the optimal dosage of IL‐2 is vital in these investigations.

## AUTHOR CONTRIBUTIONS

**Yonaton Zarbiv:** Writing – original draft (lead); writing – review and editing (lead). **Arielle Jacover:** Conceptualization (supporting); writing – original draft (equal); writing – review and editing (equal). **Keidar Haran Tal:** Formal analysis (equal); visualization (equal). **Shiri Klein:** Methodology (equal); project administration (equal). **Shani Breuer:** Project administration (equal). **Ronen Durst:** Investigation (supporting). **Batia Avni:** Investigation (supporting). **Sigal Grisariu:** Investigation (supporting). **Polina Stepensky:** Investigation (equal); writing – review and editing (equal). **Michal Lotem:** Conceptualization (equal); methodology (equal); project administration (equal). **Ofra Maimon:** Conceptualization (equal); investigation (equal); supervision (equal); writing – review and editing (equal). **Tamar Yablonski‐Peretz:** Conceptualization (equal); formal analysis (equal); investigation (equal); methodology (equal); project administration (equal); writing – original draft (equal); writing – review and editing (equal).

## CONFLICT OF INTEREST STATEMENT

The authors have stated explicitly that there are no conflicts of interest in connection with this article.

## ETHICS STATEMENT

The study was conducted at the Sharret Institute of Oncology, after approval by the Institutional review board (0394‐20‐HMO).

## INFORMED CONSENT STATEMENT

Informed patient consent was obtained for genetic analysis for this study. Expressed consent for publication was obtained by the deceased next of kin for publication and use of images.

## Data Availability

The data that support the findings of this study are available on request from the corresponding author. The data are not publicly available due to privacy or ethical restrictions.
